# Associations between partial sickness benefit and disability pensions: initial findings of a Finnish nationwide register study

**DOI:** 10.1186/1471-2458-10-361

**Published:** 2010-06-23

**Authors:** Johanna Kausto, Lauri Virta, Ritva Luukkonen, Eira Viikari-Juntura

**Affiliations:** 1 Centre of Expertise for Health and Work Ability, Finnish Institute of Occupational Health, Helsinki, Finland; 2 Social Insurance Institution, Research Department, Turku, Finland; 3 Statistical Services, Finnish Institute of Occupational Health, Helsinki, Finland

## Abstract

**Background:**

Timely return to work after longterm sickness absence and the increased use of flexible work arrangements together with partial health-related benefits are tools intended to increase participation in work life. Although partial sickness benefit and partial disability pension are used in many countries, prospective studies on their use are largely lacking. Partial sickness benefit was introduced in Finland in 2007. This register study aimed to investigate the use of health-related benefits by subjects with prolonged sickness absence, initially on either partial or full sick leave.

**Methods:**

Representative population data (13 375 men and 16 052 women either on partial or full sick leave in 2007) were drawn from national registers and followed over an average of 18 months. The registers provided information on the study outcomes: diagnoses and days of payment for compensated sick leaves, and the occurrence of disability pension. Survival analysis and multinomial regression were carried out using sociodemographic variables and prior sickness absence as covariates.

**Results:**

Approximately 60% of subjects on partial sick leave and 30% of those on full sick leave had at least one recurrent sick leave over the follow up. A larger proportion of those on partial sick leave (16%) compared to those on full sick leave (1%) had their first recurrent sick leave during the first month of follow up. The adjusted risks of the first recurrent sick leave were 1.8 and 1.7 for men and women, respectively, when subjects on partial sick leave were compared with those on full sick leave. There was no increased risk when those with their first recurrent sick leave in the first month were excluded from the analyses. The risks of a full disability pension were smaller and risks of a partial disability pension approximately two-fold among men and women initially on partial sick leave, compared to subjects on full sick leave.

**Conclusions:**

This is the first follow up study of the newly adopted partial sickness benefit in Finland. The results show that compared to full sick leave, partial sick leave - when not followed by lasting return to work - is more typically followed by partial disability pension and less frequently by full disability pension. It is anticipated that the use of partial benefits in connection with part-time participation in work life will have favourable effects on future disability pension rates in Finland.

## Background

Timely return to work after sickness absence and the use of partial health-related social security benefits are means aimed at cutting down the increased public expenditure caused by sickness. From the life-course perspective, timely or partial return to work may increase the time of an individual's lifespan spent in the labour force [1]. This is important, as there has been a long-term decline in annual time spent in paid work in many countries [2]. In addition, research evidence shows that lengthy periods of sickness absence are associated with an increased risk of permanent disability and disability pension [e.g., [3-5]]. Higher incidence of disability pensions is associated with lower effective retirement age (average age at which persons 40 and over leave labour force) in a population. Postponement of retirement is one of the key targets of the current economic and social policy in many countries.

All citizens of Finland aged 16 to 67 are eligible for compensated sick leave in cases of work incapacity due to illness. Employers continue paying salary over a 'waiting period', which comprises the day on which the illness begins plus the following nine weekdays. After this period, the Social Insurance Institution of Finland (SII) starts paying full sickness benefit. The maximum length of the compensation is 300 weekdays in two years (per disease). In 2008, the SII granted approximately 363 000 full sickness benefit periods [6]. The current cost level of full sickness benefit in Finland is about the same as a decade ago. The economic recessions in Finland (in the 1990s and 2008-2009) have to some extent decreased the use of the benefit and the expenses related to it.

Partial sickness benefit was introduced in Finland in 2007. It can be granted to employees or self-employed people between the age of 16 and 67. They must be unable to work and thereby entitled to full sickness benefit. Voluntary return to work on a part-time basis must not compromise their recovery. They must have been working full-time before the onset of work incapacity, and have been paid the regular full sickness benefit for at least 60 working days without interruption immediately before receiving the partial sickness benefit. Partial sick leave entails combining partial salary with a partial benefit, working part-time performing one's usual or modified work tasks. Working hours and salary must be cut down to 40 - 60% of the regular. The minimum duration of partial sick leave is 12 weekdays and maximum duration 72 weekdays within a period of two years. So far it has been used less than expected; only around 1 900 partial sickness benefit periods were granted by the SII in 2008 [6].

In cases of work disability, a partial or full pension can be granted either indefinitely or for a specified period. The Finnish pension scheme consists of both an earnings-related pension and a national pension, which covers the minimum income. A partial disability pension can be granted on the basis of earnings-related pensions. In 2008, 28 600 subjects retired on a disability pension in Finland [7]. Of the earnings-related pensions, 44% were temporary and 16% were partial [8]. Roughly 70% of those on partial disability pension specifically applied for a partial pension instead of a full pension, and as many worked part-time, half of them 20 - 29 hours per week. Most of those receiving partial disability pension and working part-time continued in the same occupation and workplace as before their illness [9,10]. The rest of the receivers of partial disability pension (30%) were not available for work. In Finland, the flow into disability pensions has been rather steady, whereas the use of partial disability pensions has been increasing over the recent years. This can be considered positive, as subjects on full disability pension rarely return to work [11].

Partial sickness benefit is available in many countries, but research on its effects, especially prospective studies, is sparse [12]. The aim of the present study was to prospectively investigate the recurrence of medically certified sick leaves and the occurrence of partial and full disability pensions (as a proxy for failure of return to work or work retention) in two working populations with prolonged sickness absence; one group on full sick leave followed by partial sick leave and another on full sick leave. The study was carried out using comprehensive register information regarding Finnish citizens, their medically certified sickness absence and the disability pensions granted.

## Methods

### Data and population

Population data were obtained from the SII's national sickness insurance registers. The registers cover all medically certified and compensated sick leaves in Finland that last for over ten days. The initial sample of the recipients of partial sickness benefit (n = 1 048) comprised all Finnish citizens whose partial sick leave (from here on referred to as baseline sick leave) had ended between 1 May and 31 December 2007. We restricted the sample to the four major diagnostic entities according to the International Classification of Diseases, version 10 (ICD10): mental and musculoskeletal disorders, traumas, and tumours.

Accordingly, the initial sample of recipients of full sickness benefit (the initial referent group, n = 37 817) included all subjects with the abovementioned diagnoses, whose full sick leave (later referred to as baseline sick leave) had ended within the same calendar period with a continuous phase of at least 60 days of payment at the end of the respective sick leave period.

One individual who had received partial sickness benefit died before the end of the baseline sick leave period and was excluded from the final sample. In the full sick leave group, 448 subjects died before the end of the baseline, two subjects turned 68 years of age during baseline sick leave, 183 subjects were granted disability pension directly after baseline sick leave, and 8 804 subjects were not working before baseline sick leave. This total of 9 437 subjects was excluded from the final sample. The final study sample thus consisted of 1 047 subjects on partial sick leave and 28 380 subjects on full sick leave.

All the subjects were followed from the end of the full or partial sick leave period at baseline until they turned 68, died or the end of the follow up on 31 May 2009. Information on disability pensions was obtained from a register maintained jointly by the SII and the Finnish Centre for Pensions. The register provided information on whether an individual received (yes/no) disability pension (full or partial, temporary or permanent) on the last day of 2007 and 2008. If a disability pension had been granted, the follow up of an individual was terminated at the end of the year in question. Unfortunately, the registers available in this study did not provide information on the employment status (possible unemployment) of either group.

### Outcome measures

We present descriptive data regarding the occurrence of sick leaves and disability pensions. Multivariable models were used to take into account the effects of covariates. Data on days of payment and diagnoses for compensated sick leaves (any type and cause) during follow up were obtained from the national registers. We categorized the diagnoses according to the ICD10, and constructed variables describing time to the first recurrent sick leave (for any cause) and the number of sickness absence episodes. In the multinomial regression analysis, the outcome was occurrence of disability pension (1 = no disability pension, 2 = partial disability pension, 3 = full disability pension).

### Explanatory measures

Information on gender, date of birth (to calculate age at the end of the baseline sick leave), area of residence (insurance district), annual gross income in 2006, type (partial or full sick leave), cause (diagnostic codes according to ICD10), length (days) of the baseline sick leave, and length of previous sickness absence (total number of compensated full sickness benefit days in two preceding years) were obtained from the sickness insurance registers.

### Statistical methods

Survival analysis (Kaplan-Meier with Log Rank-test, and Cox proportional hazards regression analysis) was applied to investigate the occurrence of compensated sick leave periods and hazard ratios with 95% confidence intervals for the first recurrent sick leave. We conducted repeated measures survival analysis in order to look into the total number of sick leaves, and to take into account the possibility of an individual being granted multiple sick leaves during follow up. Multinomial regression analysis was run in order to calculate the odds ratios for full and partial disability pensions. We investigated both crude associations and associations adjusted for age, diagnostic group, area of residence, income, length of baseline sick leave period, and previous sickness absence. Subjects who were on full sick leave at baseline, constituted the reference category in the Cox and multinomial regression models. To examine whether the incidence of sick leave and the survival functions in the studied groups were modified by the diagnostic group, stratified Kaplan Meier analyses were performed. The analyses were conducted in both all cases, and in those with no recurrences during the first 30 days of follow up. This was done in order to first have a look at all the subjects in the study samples, and then to look at those subjects who supposedly succeeded in the return to work after baseline sick leave. All analyses were stratified by gender, since men and women differ regarding sickness absence and disability pensions. The analyses were performed using statistical software programme SPSS, version 15.0 and SAS, version 9.1 (SAS Institute Inc., Cary, NC).

## Results

### Characteristics of study samples at baseline

Table 1 shows the distribution of the study sample into partial and full sick leave groups and the categories of the explanatory variables at baseline when stratified by gender. Of the subjects on partial sick leave, 72% were women; the corresponding proportion among those on full sick leave was 54%. The proportion of subjects aged 45 to 54 was somewhat higher, and the proportion of those aged over 55 was lower in the partial compared to the full sick leave group. The income level of subjects on partial sick leave was somewhat higher than that of those on full sick leave.

**Table 1:** Characteristics of participants at baseline.

	Men (n = 13 375)				Women (n = 16 052)			
	**Partial sick leave** **(n = 293)**		**Full sick leave** **(n = 13 082)**		**Partial sick leave** **(n = 754)**		**Full sick leave** **(n = 15 298)**	
	**n**	**%**	**n**	**%**	**n**	**%**	**n**	**%**

**Age, years**								
- 34	45	15.4	2 279	17.5	74	9.9	2 161	14.1
35 - 44	70	23.9	2 592	19.8	170	22.5	2 811	18.4
45 - 54	110	37.5	4 153	31.7	338	44.8	5 108	33.4
55 - 67	68	23.2	4 058	31.0	172	22.8	5 218	34.1
Mean (SD)	46.0 (9.8)		46.7 (11.3)		47.3 (8.6)		47.9 (10.8)	
								
**Gross income, €/year**								
- 30 000	164	57.3	9 105	69.6	594	81.0	13 420	87.7
30 001 - 60 000	111	38.8	3 684	28.2	126	17.2	1 773	11.6
60 001 -	11	3.9	293	2.2	13	1.8	105	0.7
Mean (SD)	30 944 (14 104)		26 254 (14 986)		25 633 (9 964)		21 286 (10 795)	
								
**Main diagnostic groups**								
Mental disorders	117	39.9	2 975	22.7	321	42.5	4 694	30.7
Musculoskeletal disorders	118	40.3	6 073	46.4	290	38.5	7 020	45.9
Tumours	17	5.8	778	6.0	86	11.4	1 643	10.7
Traumas	41	14.0	3 256	24.9	57	7.6	1 941	12.7
								
**Insurance district**								
Northern	39	13.3	1 697	13.0	104	13.8	1 901	12.5
Western	65	22.2	1 737	13.3	109	14.5	1 976	12.9
Eastern	26	8.9	2 001	15.3	97	12.9	2 189	14.3
South-Western	69	23.5	3 154	24.1	168	22.3	3 689	24.1
Southern	94	32.1	4 493	34.3	276	36.5	5 543	36.2
								
**Previous sickness absence, days **(in two years prior to baseline sick leave)								
0 - 59	0	0.0	11 447	87.5	0	0.0	13 154	86.0
60 - 99	68	23.3	778	6.0	189	25.2	978	6.4
100 - 300	224	76.7	855	6.5	561	74.8	1 166	7.6
Mean (SD)	157.6 (64.3)		19.4 (40.9)		152.9 (64.0)		22.6 (43.3)	
								
**Baseline sick leave, days**								
4 - 59	142	48.5	0	0.0	400	53.1	0	0.0
60 - 72	151	51.5	2 369	18.1	354	46.9	2 626	17.2
73 - 150	0	0.0	4 825	36.9	0	0.0	5 692	37.2
151 - 300	0	0.0	5 888	45.0	0	0.0	6 980	45.6
Mean (SD)	55.6 (18.3)		160.5 (85.2)		53.2 (19.1)		159.4 (82.8)	
								
**Previous sickness absence + baseline sick leave, days**								
60 - 99	7	2.4	3 799	29.0	23	3.1	4 133	27.0
100 - 150	62	21.2	2 317	17.7	159	21.2	2 740	17.9
151 - 300	185	63.4	6 927	53.0	491	65.4	8 356	54.6
301 - 429	38	13.0	37	0.3	77	10.3	69	0.5
Mean (SD)	213.3 (69.1)		179.9 (89.3)		206.2 (69.2)		182.0 (87.6)	

A mental disorder was the cause for partial sick leave more often than for full sick leave. Depression (F32) was the most common single diagnosis in all subgroups, the proportion of depression in all diagnoses being 8% in the group of men on full sick leave, and 14% in the group of men on partial sick leave. The corresponding proportions among women were 11% and 15%, respectively. Among women on partial sick leave, the second most common single diagnosis was recurrent depression (F33), at 6%. Among men on full sick leave, the second most common diagnosis was shoulder lesions (M75), at 4%, and among men on partial sick leave, other intervertebral disc disorders (M51), at 8%. The second most common diagnoses among women on full sick leave were other intervertebral disc disorders (M51) and breast cancer (C50), covering 5% and 3% of all the diagnoses in this group, respectively.

At baseline, partial sick leave periods varied from 4 to 72 days and full sick leave periods from 60 to 300 days. The range of previous sickness absence (total number of compensated full sickness benefit days in two preceding years) was from 60 to 299 days in the partial sick leave group and from 0 to 255 days in the full sick leave group. Adding up the number of sickness benefit days at baseline to sickness benefit days in the two previous years revealed no major difference in total sickness absence between the two sick leave groups (the mean of the total sickness absence being 208 days among those on partial sick leave, and 181 days among those on full sick leave).

### Occurrence of sick leaves during follow up

The follow up time varied from 0 to 25 months. In the partial sick leave and full sick leave groups, 19 and 602 subjects died during follow up, respectively. In the full sick leave group, 30 subjects turned 68 before the end of the follow up.

A total of 9719 subjects; 58% of those on partial sick leave at baseline (611 subjects) and 32% of those on full sick leave (9108 subjects), had at least one medically certified sick leave (any cause) lasting more than ten days during the follow up. Time to the first recurrent sick leave (for any cause) varied from 0 to 25 months. In the partial sick leave and full sick leave groups, 16% and 1% of the subjects, respectively, had their first recurrent episode of sick leave during the first 30 days of follow up, most of them immediately.

Table 2 presents the recurrence of medically certified sick leaves in the total study sample during the entire follow up period. The mean time to the first recurrent sick leave (censored data information taken into account) was shorter, and the incidence rate of the recurrences higher among those initially on partial sick leave.

**Table 2:** Incidence rate and latency of recurrence of medically certified sick leaves.

	Men		Women	
	**Partial sick leave** **(n = 293)**	**Full sick leave** **(n = 13 082)**	**Partial sick leave** **(n = 754)**	**Full sick leave** **(n = 15 298)**

Mean time (months) to the first recurrent sick leave (95% CI) (Kaplan - Meier analysis)	14.5 (13.3 - 15.7) (n of events = 154)	19.3 (19.1 - 19.5) (n of events = 3 890)	13.5 (12.8 - 14.2) (n of events = 457)	18.5 (18.3 - 18.6) (n of events = 5 218)
p Log Rank	0.000		0.000	
Total no of sickness absence days	12 463	275 141	33 592	345 484
Person months	5 745	227 598	15 249	265 830
Incidence Rate (recurrent sick leaves/person month)	0.04	0.03	0.05	0.03

The incidence of recurrent sick leaves was also investigated in a subsample of the subjects with no recurrences during the first 30 days of follow up. The results showed that the differences between the partial sick leave and referent groups were diminished. Among men, the mean time (months) to the first recurrent sick leave (95% CI) in these two groups was 17.8 (16.7 - 19.0) and 19.5 (19.3 - 19.7), respectively, and incidence rates (recurrent sick leaves/person month) 0.03 and 0.03, respectively. Among women, the corresponding values were 16.0 (15.2 - 16.7) and 18.8 (18.6 - 18.9), and 0.04 and 0.03, respectively.

### Stratified analyses

The Kaplan-Meier analyses were carried out stratified by the diagnosis of baseline sick leave. The differences in the mean time to the first sick leave between the partial sick leave and referent groups did not change significantly when studied according to diagnostic group, taking all the cases into account. The analysis was then run in the subsample of the subjects with no recurrences during the first 30 days of follow up. Among men with a mental disorder as the cause for baseline sick leave, there was no significant difference in the mean time to the first sick leave between the partial sick leave (mean 18.6 months, 95% CI 16.8 - 20.3) and the referent group (mean 19.5 months, 95% CI 19.2 - 19.9).

The analyses were then performed among those who were initially on partial sick leave in order to find out whether the time to first sick leave differed between the diagnostic groups. There were no significant differences between the diagnostic groups, either when all the cases were looked into or when the analysis was run in the subsample of subjects with no recurrences during the first 30 days of follow up.

### Associations between type of sick leave and sickness absence

Compared to the referent group, both men and women in the partial sick leave group had an increased crude and adjusted risk of first recurrent sick leave (Table 3). The crude risk of first recurrent sick leave was roughly two-fold in the partial sick leave group. When adjusted for age, diagnostic group at baseline, insurance district, gross income, length of previous sickness absence, and length of baseline sick leave, the hazard ratios decreased to 1.8 (1.4 - 2.3) and 1.7 (1.4 - 2.0), respectively. The crude risk of cumulative number of recurrences was 1.3-fold in the partial sick leave group compared to the full sick leave group. After adjustment for the other characteristics of the participants, the hazard ratios remained at the same level.

**Table 3:** Hazard Ratios for medically certified recurrent sick leaves (Cox regression and repeated measures survival analysis).

	Time to first recurrence (any cause)		Cumulative no of all recurrences (any cause)	
****	**Crude** **Hazard Ratio** **(95% CI)**	**Adjusted** **Hazard Ratio** **(95% CI)^1^**	**Crude** **Hazard Ratio** **(95% CI)**	**Adjusted** **Hazard Ratio** **(95% CI)^1^**

Men (n = 13 365)				
Partial sick leave	1.9 (1.6 - 2.3)	1.8 (1.4 - 2.3)	1.3 (1.1 - 1.4)	1.3 (1.1 - 1.6)
Full sick leave	1.0	1.0	1.0	1.0
				
Women (n = 16 027)				
Partial sick leave	2.0 (1.8 - 2.2)	1.7 (1.4 - 2.0)	1.3 (1.2 - 1.4)	1.4 (1.3 - 1.5)
Full sick leave	1.0	1.0	1.0	1.0

^1^Adjusted for age, diagnostic group at baseline, insurance district, gross income, length of previous sickness absence, and length of baseline sick leave.

The associations were also studied in the subsample of the subjects with no recurrent sick leaves during the first 30 days of follow up. The crude risk of first recurrent sick leave was somewhat higher in the partial sick leave group compared with the full sick leave group. When adjusted for background factors, there was no significant difference between the sick leave groups. Men on partial sick leave had a 1.3-fold (1.1 - 1.6) crude risk of first recurrent sick leave compared to men on full sick leave, the adjusted hazard ratio being 0.8 (0.7 - 1.1). Women on partial sick leave had a 1.5-fold crude risk of first recurrent sick leave compared to women on full sick leave. When adjusted for background factors, the hazard ratio was 1.1 (0.9 - 1.2). The risks of the cumulative number of recurrences did not change when the analyses were carried out in this subsample.

### Disability pensions

**Figure 1:**
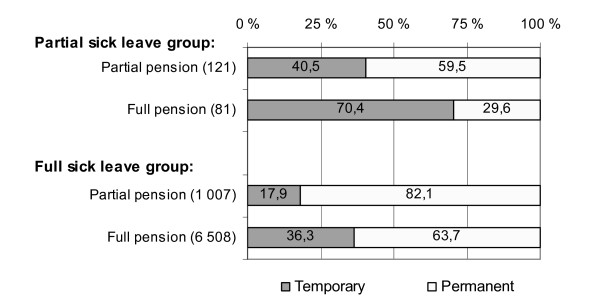
**Temporary and permanent disability pensions**.

On 31 December 2007, 69 subjects on partial sick leave at baseline and 5 855 subjects on full sick leave received partial or full disability pension. A year later, the respective figures in the two groups were 202 and 7 515. Table 4 presents the prevalences of partial and full disability pension on 31 December 2008 by gender and type of baseline sick leave. Full disability pension was more prevalent in the full sick leave group among both genders, whereas partial disability pension was more prevalent in the partial sick leave group, among both men and women. In the partial sick leave group, 48% of all the pensions were permanent. The respective share in the full sick leave group was 66% (Figure 1). In all, among those on partial sick leave, more than 90% and among those on full sick leave, 77% of the subjects were available for work (on full or part-time basis) at the end of 2008.

**Table 4:** Prevalence of disability pension (n, percentage of the subjects in the subgroup) (31.12.2008).

		Partial pension		Full pension		All pensions	
	**(n)**	**(no of cases)**	**%**	**(no of cases)**	**%**	**(no of cases)**	**%**

Men							
Partial sick leave	(283)	(27)	9.5	(28)	9.9	(55)	19.4
Full sick leave	(12 817)	(319)	2.5	(3 060)	23.9	(3 379)	26.4
							
Women							
Partial sick leave	(748)	(94)	12.6	(53)	7.1	(147)	19.7
Full sick leave	(15 092)	(688)	4.6	(3 448)	22.8	(4 136)	27.4
							
All							
Partial sick leave	(1 031)	(121)	11.7	(81)	7.9	(202)	19.6
Full sick leave	(27 909)	(1 007)	3.6	(6 508)	23.3	(7 515)	26.9

### Associations between characteristics of participants and partial and full disability pensions

Table 5 and Table 6 present crude and adjusted odds ratios with 95% CIs for the associations between the characteristics of the participants, and partial and full disability pension on 31 December 2008. The adjusted risks for full disability pension decreased to 0.2-fold and for partial disability pension increased to roughly 2.0-fold for both men and women initially on partial sick leave, compared to subjects on full sick leave. Higher age was very strongly associated with subsequent partial disability pension, among both genders. An increased risk for partial disability pension was also found for mental and musculoskeletal disorders, and a higher number of sickness absence days. There was no association between the income level or the area of residence and occurrence of partial disability pension. In both genders, higher age, mental and musculoskeletal disorders, and a higher number of sickness absence days, as well as tumours, lower income and northern area of residence in men, were associated with a subsequent full disability pension.

**Table 5:** Odds Ratios for associations between characteristics of participants and disability pensions among men (multinomial regression analysis).

Men (n = 13 100)					
		**Partial disability pension**		**Full disability pension**	
	**n**	**Odds Ratio**	**95%CI**	**Odds Ratio**	**95%CI**

*Crude*					
					
**Type of sick leave**					
Full sick leave	12 817	1.0		1.0	
Partial sick leave	283	3.5	(2.3 - 5.3)	0.4	(0.3 - 0.6)
					
*Fully adjusted*					
					
**Type of sick leave**					
Full sick leave	12 815	1.0		1.0	
Partial sick leave	276	2.0	(1.2 - 3.2)	0.2	(0.1 - 0.3)
**Age, years**					
- 34	2 304	1.0		1.0	
35 - 44	2 636	6.2	(1.9 - 20.8)	1.0	(0.8 - 1.2)
45 - 54	4 143	33.2	(10.5 - 104.5)	2.3	(2.0 - 2.8)
55 - 67	4 008	46.3	(14.6 - 146.2)	5.7	(4.8 - 6.8)
**Diagnostic group**					
Traumas	3 266	1.0		1.0	
Mental disorders	3 045	3.2	(2.1 - 4.7)	3.4	(2.9 - 3.9)
Musculoskeletal disorders	6 147	2.4	(1.7 - 3.5)	1.5	(1.3 - 1.8)
Tumours	633	1.0	(0.5 - 2.0)	1.5	(1.1 - 1.9)
**Gross income, €/year**					
60 001 -	295	1.0		1.0	
30 001 - 60 000	3 707	0.8	(0.4 - 1.6)	1.5	(1.1 - 2.2)
- 30 000	9 089	1.1	(0.6 - 2.2)	1.9	(1.4 - 2.8)
**Insurance district**					
Southern	4 490	1.0		1.0	
Northern	1 702	1.3	(0.9 - 1.9)	1.3	(1.1 - 1.5)
Western	1 757	1.2	(0.8 - 1.7)	0.9	(0.8 - 1.1)
Eastern	1 994	0.9	(0.6 - 1.2)	1.1	(1.0 - 1.3)
South - Western	3 148	1.2	(0.9 - 1.7)	1.0	(0.9 - 1.1)
**Previous sickness absence + baseline sick leave, days**					
60 - 99	3 772	1.0		1.0	
100 - 150	2 350	2.2	(1.4 - 3.4)	1.6	(1.2 - 2.2)
151 - 300	6 899	4.8	(3.4 - 6.9)	23.6	(19.1 - 29.1)
301 - 429	70	11.2	(4.6 - 27.5)	26.6	(14.2 - 50.0)

**Table 6:** Odds Ratios for associations between characteristics of participants and disability pensions among women (multinomial regression analysis).

Women (n = 15 840)					
		**Partial disability pension**		**Full disability pension**	
	**n**	**Odds Ratio**	**95%CI**	**Odds Ratio**	**95%CI**

*Crude*					

**Type of sick leave**					
Full sick leave	15 092	1.0		1.0	
Partial sick leave	748	2.5	(2.0 - 3.1)	0.3	(0.2 - 0.4)
					
*Fully adjusted*					
					
**Type of sick leave**					
Full sick leave	15 092	1.0		1.0	
Partial sick leave	723	1.9	(1.5 - 2.4)	0.2	(0.1 - 0.2)
**Age, years**					
- 34	2 223	1.0		1.0	
35 - 44	2 937	3.4	(1.8 - 6.6)	1.1	(0.9 - 1.3)
45 - 54	5 370	14.2	(7.8 - 26.2)	2.1	(1.8 - 2.5)
55 - 67	5 285	25.5	(13.9 - 46.8)	6.8	(5.7 - 8.0)
**Diagnostic group**					
Traumas	1 989	1.0		1.0	
Mental disorders	4 983	2.4	(1.7 - 3.3)	4.0	(3.3 - 4.9)
Musculoskeletal disorders	7 275	2.5	(1.8 - 3.3)	2.1	(1.7 - 2.5)
Tumours	1 568	0.8	(0.5 - 1.2)	1.2	(0.9 - 1.5)
**Gross income, €/year**					
60 001 -	114	1.0		1.0	
30 001 - 60 000	1 864	0.6	(0.2 - 1.6)	1.1	(0.6 - 2.1)
- 30 000	13 837	1.1	(0.4 - 2.8)	1.5	(0.8 - 2.9)
**Insurance district**					
Southern	5 711	1.0		1.0	
Northern	1 980	1.2	(0.9 - 1.5)	1.1	(1.0 - 1.3)
Western	2 060	1.0	(0.8 - 1.3)	0.8	(0.7 - 0.9)
Eastern	2 257	1.1	(0.9 - 1.4)	1.0	(0.9 - 1.2)
South - Western	3 807	0.9	(0.8 - 1.2)	0.9	(0.8 - 1.0)
**Previous sickness absence + baseline sick leave, days**					
60 - 99	4 142	1.0		1.0	
100 - 150	2 881	1.6	(1.3 - 2.1)	2.4	(1.8 - 3.0)
151 - 300	8 649	3.4	(2.7 - 4.2)	24.0	(19.6 - 29.4)
301 - 429	143	5.8	(3.2 - 10.4)	40.9	(25.2 - 66.1)

## Discussion

This study presents large register data on the medically certified sickness absence and disability pensions of two working populations with prolonged sickness absence. Compared with subjects on full sick leave, those on partial sick leave had a shorter latency of recurrence, higher incidence rate of recurrent sick leaves, and an increased risk of recurrent sick leave during the average 18 months of follow up. A larger proportion of subjects in the partial sick leave than in the full sick leave group had their first recurrent episode of sick leave immediately after baseline sick leave or during the first month of follow up. It is possible that this refers to the fact that during this initial stage of use of the new partial sickness benefit in Finland, physicians' prescriptions of partial sick leave together with assessment of work incapacity has, to a certain extent, been inadequate. Either expectations of return to work have been too optimistic, the time needed for full recovery has been underestimated, or sufficient work arrangements during partial sick leave have not been carried out at the workplace. When these subjects were excluded from the analyses, i.e., attention was paid to subjects who most probably succeeded in returning to work after the baseline sick leave, differences in the risks of the first recurrent sick leave between the two sick leave groups were eliminated. The risk of cumulative number of recurrences was still slightly higher in the partial sick leave group. There are only a few earlier prospective studies on the use of partial sick leave with diverse study designs and inconsistent findings [12]. Our findings on the recurrence of sick leave are, to a degree, in line with earlier results, indicating that subjects on partial sick leave are not likely to return to work any sooner than subjects on full sick leave. Our results are somewhat opposed to earlier results, which have shown that increased use of partial sick leave does not affect the recurrence of sick leave.

Partial and temporary pensions were more common and full pensions less common among subjects initially on partial sick leave. The adjusted risks of partial disability pension were two-fold among men and women on partial sick leave compared to subjects on full sick leave. To our knowledge, associations between partial sick leave and partial disability pension have not been previously reported. When adjusted for background factors, the use of partial sick leave was associated with a lower risk of full disability pension. The latter finding is incompatible with Norwegian studies which report no association between the increased use of partial sick leave and long-term disability, or the use of disability benefits [13,14]. In agreement with previous studies on predictors of disability pensions [3-5,15-20], we found that higher age, a mental or musculoskeletal disorder, and a higher number of sickness absence days were associated with subsequent partial or full disability pension. In addition, in line with earlier findings [17] sick leaves for mental disorders were strong predictors of full disability pension.

### Strengths and limitations of the study

The present study adds to the knowledge on the use of partial health-related benefits. The findings can be generalized for the current Finnish working population with prolonged sickness absence. We could avoid sample attrition and did not need to rely on self-reported data, as we used register-based information. As with earlier studies, registers provided only one diagnostic code per sickness absence episode, and thus co-morbidity could not be looked into [21]. This has been observed to be a common restriction of register-based studies. Moreover, the registers accessible in this study did not provide diagnoses for partial or full disability pensions, which prevented us from studying diagnosis-specific associations between sickness absence and disability pension [17].

Explanatory measures of this study were limited to the sociodemographics, diagnostic codes, and information on prior sickness absence provided by the registers. These factors have previously been shown to be important predictors of the use of health-related benefits. There are, however, a number of potential individual level and contextual predictors of sickness absence and disability pension, such as personality, family, health behaviour, occupational, organizational, and societal (structural, political or cultural) factors. Since all possible confounders can not be covered simultaneously, they constitute a possible source of bias. This study showed that subjects on either partial or full sick leave did not considerably differ from each other regarding either their sociodemographic background, or their sickness absence as a total of sickness absence days at baseline and during the two earlier years. The analyses should, however, be replicated taking into account a broader set of explanatory factors in different social contexts. It has been pointed out that the follow up of disability pensions should not be started directly after baseline sick leave period in order to avoid overestimation of the associations between these constructs [17,22]. However, the short time-frame of this study did not allow us to postpone the start of the follow up. Nonetheless, we did exclude from the analyses subjects who had been granted disability pension directly after baseline sick leave. This also calls for a replication of the analyses with a longer follow up in the future.

## Conclusions

These findings from prospective national register data on the Finnish working population with prolonged sickness absence suggest that compared to subjects on full sick leave, subjects on partial sick leave had less success in their initial return to work. However, when closer attention was paid to the subjects who best succceeded in returning to work after baseline sick leave, there was no difference in the adjusted risks of the first recurrent sick leave between the two groups. The results show that compared to full sick leave, partial sick leave - when not followed by lasting return to work - is more typically followed by partial disability pension and less frequently by full disability pension. Even if major confounding in the studied associations is not likely, the analyses should be replicated with a larger set of adjusted factors. If the relationships detected in this study prove to be robust, the use of partial sickness absence might in part slow down the flow into permanent and full disability.

## Ethical considerations

Ethical approval was not necessary, since we used only encrypted register data and did not contact the unidentifiable study subjects.

## Competing interests

The authors declare that they have no competing interests.

## Authors' contributions

JK and LV planned the study design, were responsible for drawing the sample from the national registers and prepared the manuscript draft. JK and RL planned and carried out the statistical analyses. EVJ participated in planning the study design and analyses, and commented on the manuscript draft. All authors have read and approved the final manuscript.

## Pre-publication history

The pre-publication history for this paper can be accessed here:

http://www.biomedcentral.com/1471-2458/10/361/prepub

## Ackowledgements

We thank Ms Minna Aalto of the Social Insurance Institution and Ms Tuula Kyyrä of the Finnish Centre for Pensions for management of the register sample.
